# Global forest typology at 10-meter resolution for forest and land-use monitoring

**DOI:** 10.1038/s41597-026-08299-5

**Published:** 2026-10-21

**Authors:** Maxim Neumann, Anton Raichuk, Peter Potapov, Myroslava Lesiv, Matthew Overlan, Mélanie Rey, Ravindran Rajakumar, Michelangelo Conserva, Radost Stanimirova, Michelle J. Sims, Sarah Carter, Elizabeth Goldman, Yuchang Jiang, Linus Scheibenreif, Ivelina Georgieva, Maria Shchepashchenko, Steffen Fritz, Nicholas Clinton, Charlotte Stanton, Dan Morris, Drew Purves

**Affiliations:** 1 Google DeepMind, Zurich, Switzerland; 2 World Resources Institute, Washington, DC USA; 3 International Institute for Applied Systems Analysis, Laxenburg, Austria; 4 Google DeepMind, London, UK; 5 Google Research, Mountain View, CA USA; 6 World Resources Institute, The Hague, Netherlands; 7 University of Zurich, Zurich, Switzerland; 8 ETH, Zurich, Switzerland; 9 Google, Mountain View, CA USA

## Abstract

Distinguishing forest types—primary, naturally regenerating, planted, and plantation forests—from agricultural tree crops and other land uses is essential for carbon accounting, biodiversity assessment, conservation planning, and supply-chain regulation. However, no existing global dataset resolves this typology at high spatial resolution. We present the Forest Typology (ForTy) v1 dataset, a global 10-meter resolution baseline map for 2020 that classifies all land into six categories aligned with FAO and EU Deforestation Regulation (EUDR) definitions: Primary Forest, Naturally Regenerating Forest, Planted Forest, Plantation Forest, Tree Crops and Agroforestry, and Other Land. A cascaded deep learning pipeline, trained on 1.7 million globally distributed samples, generates per-class relative confidence maps from geospatial satellite embeddings by combining weakly supervised learning with active learning. Independent validation against 8,190 expert-labeled stratified random sites yields an overall accuracy of 90.2% for the six-class scheme, 94.8% for natural forest classification, and 95.5% for forest/non-forest classification.

## Background & Summary

Forests cover approximately 31% of the world’s land area, store roughly 662 gigatonnes (Gt) of carbon^1^, and harbor the majority of terrestrial biodiversity^2^. Yet the ecological and economic value of a forest stand depends on its type and management history^3^: for example, primary forests differ from planted and plantation forests and agricultural tree crops by orders of magnitude in carbon density, species richness, and importance to indigenous peoples and local communities, as well as in legal status. Carbon accounting^4^, biodiversity assessment, REDD+ verification, and land-use monitoring all require classification that goes beyond biophysical tree cover presence to distinguish natural forests from planted forests, plantation forests, and agricultural tree crops. Such typological detail is also critical for assessing progress toward the Kunming-Montreal Global Biodiversity Framework’s “30 × 30” target, which aims to protect 30% of the Earth’s land area by 2030^5,6^. Since primary and some naturally regenerating forests harbor the highest biodiversity value, identifying their extent and spatial distribution at the national level is a prerequisite for prioritizing protection of remaining natural forests. In the absence of a globally consistent, high-resolution forest typology, forest monitoring efforts must fall back on binary tree cover as a proxy^7^—unable to report what forest or tree cover types are being lost or gained.

This gap carries growing policy consequences. The European Union’s Regulation on Deforestation-free Products (EUDR) mandates that commodities entering the EU market are both deforestation-free and forest degradation-free, using December 31, 2020 as the baseline^8^. The EUDR distinguishes “deforestation” (conversion of forest to agricultural use) from “forest degradation” (structural changes within the forest land use, e.g., conversion of primary forests to plantations). Monitoring these dynamic processes requires a *static typological baseline* that records the forest type at each location at the reference date, not merely whether trees are present^8,9^. Such a baseline classifies forest *state* at a single reference date; deforestation and degradation can then be assessed through subsequent change detection against this reference. The EUDR further distinguishes forest from agricultural land use, explicitly excluding tree crop plantations and agroforestry systems from its definition of forest^10^. Assessing deforestation risk under the EUDR thus requires a mapping system that can both classify forests by type and reliably separate tree crops from timber plantations—a distinction often invisible in common high-resolution satellite imagery and tree cover maps. Beyond the EUDR, the Tropical Forest Forever Facility (TFFF), which offers payments per hectare of standing tropical forest, will similarly need to exclude plantations and tree crops from eligible forest area to avoid crediting agricultural tree cover^11^; moreover, a forest typology that separates primary from secondary forest could support more comprehensive degradation monitoring beyond fire-based proxies in future TFFF methodology iterations. Recent analyses have highlighted that existing geospatial datasets fall short of the thematic detail required for these applications^12^.

Several global and regional datasets have characterized parts of this typological spectrum, but none resolve the full multi-class typology at scale. Primary forest extent can only be approximated from tropical-only or coarse-resolution products^13,14^. The Spatial Database of Planted Trees (SDPT)^15,16^ compiles known plantation boundaries from government, academic, and NGO sources, but its coverage remains geographically incomplete and temporally inconsistent. No global map separates agricultural tree cover (e.g., oil palm, rubber, cocoa) from forest. Building on earlier work by Schulze *et al*.^17^, who modeled the global distribution of forest management types from national statistics using soil, climatic, terrain, and socio-economic predictors, Lesiv *et al*. published the Global Forest Management (GFM) map for 2015 at 100 m resolution^18^, classifying forests into intact, managed (with or without logging), and planted categories based on crowd-sourced visual interpretation. More recently, the Joint Research Centre (JRC) of the European Commission produced the Global Forest Cover (GFC2020) map^19,20^ and an associated Global Forest Types 2020 (GFT2020) product^21^ to directly support the EUDR. The GFC2020 map provides a forest/non-forest binary at 10 m resolution, while the GFT2020 distinguishes primary, naturally regenerating, and planted forests^22^. The Natural Forests of the World (NFW) 2020 dataset^23^ is a global 10 m map distinguishing natural forest from all other land cover. At regional scale, the Tropical Moist Forest (TMF) dataset^24^ provides long-term monitoring (1990–2025) of tropical humid forest disturbance and degradation at 30 m resolution, distinguishing intact from degraded and deforested areas. Dalagnol *et al*.^25^ mapped tropical forest degradation using Planet NICFI imagery and deep learning, demonstrating the potential of high-resolution data for disturbance detection. Jiang *et al*.^26^ produced the first 10 meter resolution tree crop map for the South American continent. However, none of these products resolve the full multi-class typology—including primary and secondary forest sub-types, planted versus plantation forest, and tree crops—at global scale. We note that the upstream products listed above use heterogeneous and often incompatible definitions of forest, forest type, and management (ranging from biophysical tree cover thresholds to management-based classifications to spectral-temporal trajectories), and we do not adopt any single product’s labels as-is. Instead, our training pipeline combines, adapts, and cross-validates these sources against our FAO/EUDR-aligned class definitions.

The challenge is three-fold. First, forest typology is fundamentally a land *use* classification, yet satellite remote sensing observes land *cover*. FAO definitions of forest types (primary, naturally regenerating, planted, plantation) are based on management history and intent rather than single-year observable biophysical properties^27^. A mature planted forest of native species may be spectrally indistinguishable from a naturally regenerated stand of the same age and composition; conversely, a forest that has burned (or an unstocked timber plantation after harvest) remains forest by definition, yet appears as bare ground and tree cover loss in satellite-derived change maps. Second, planted forests, plantation forests, and tree crops can share similar spectral and structural signatures, particularly in tropical regions where canopy closure is rapid^28^. Third, the expression of each class varies widely across biomes and management traditions, demanding exhaustive and geographically diverse training data to capture this variability in a single globally consistent model.

**Fig. 1:**
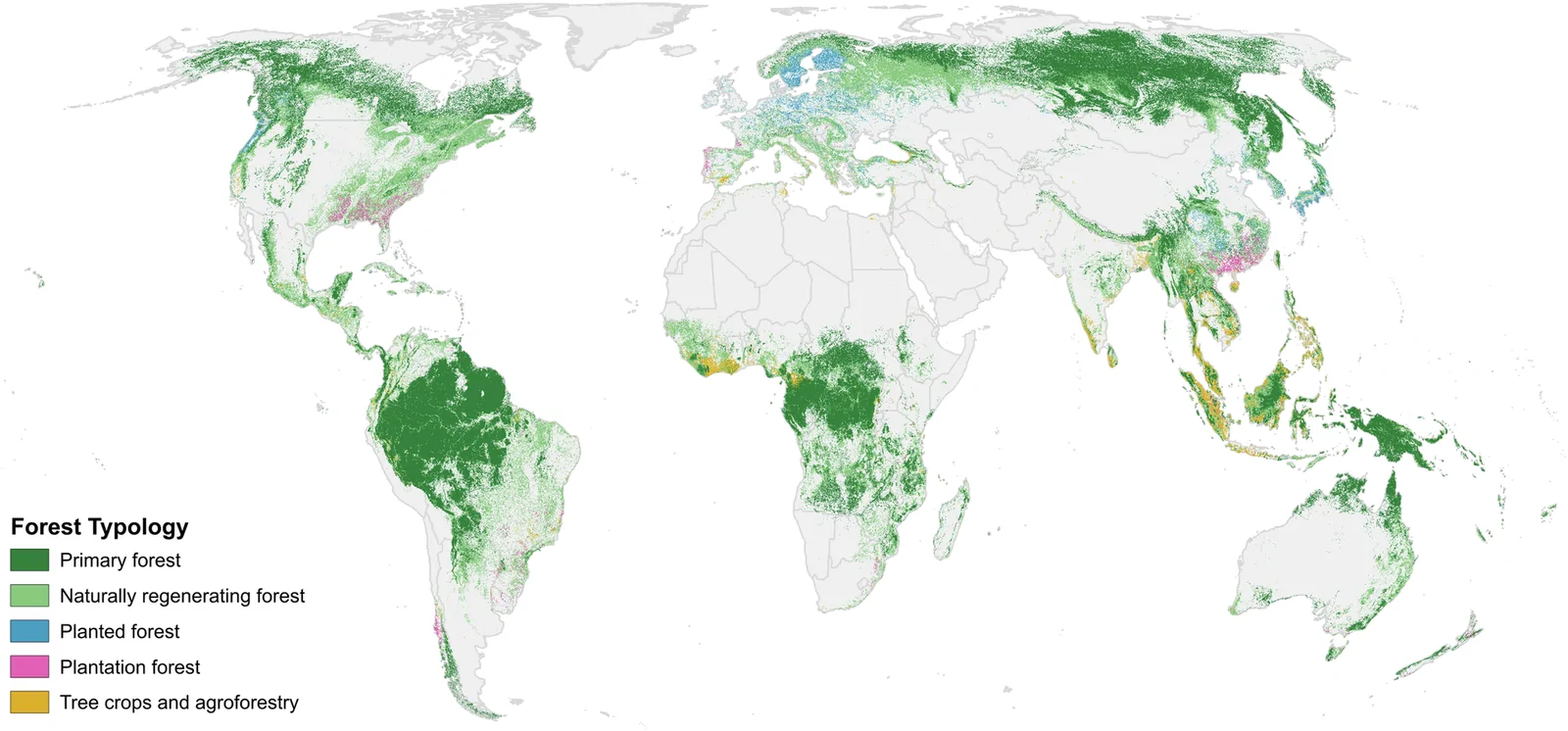
Forest Typology 2020 (v1) in Equal Earth projection showing the majority class within each 1 km^2^ cell.

**Fig. 2:**
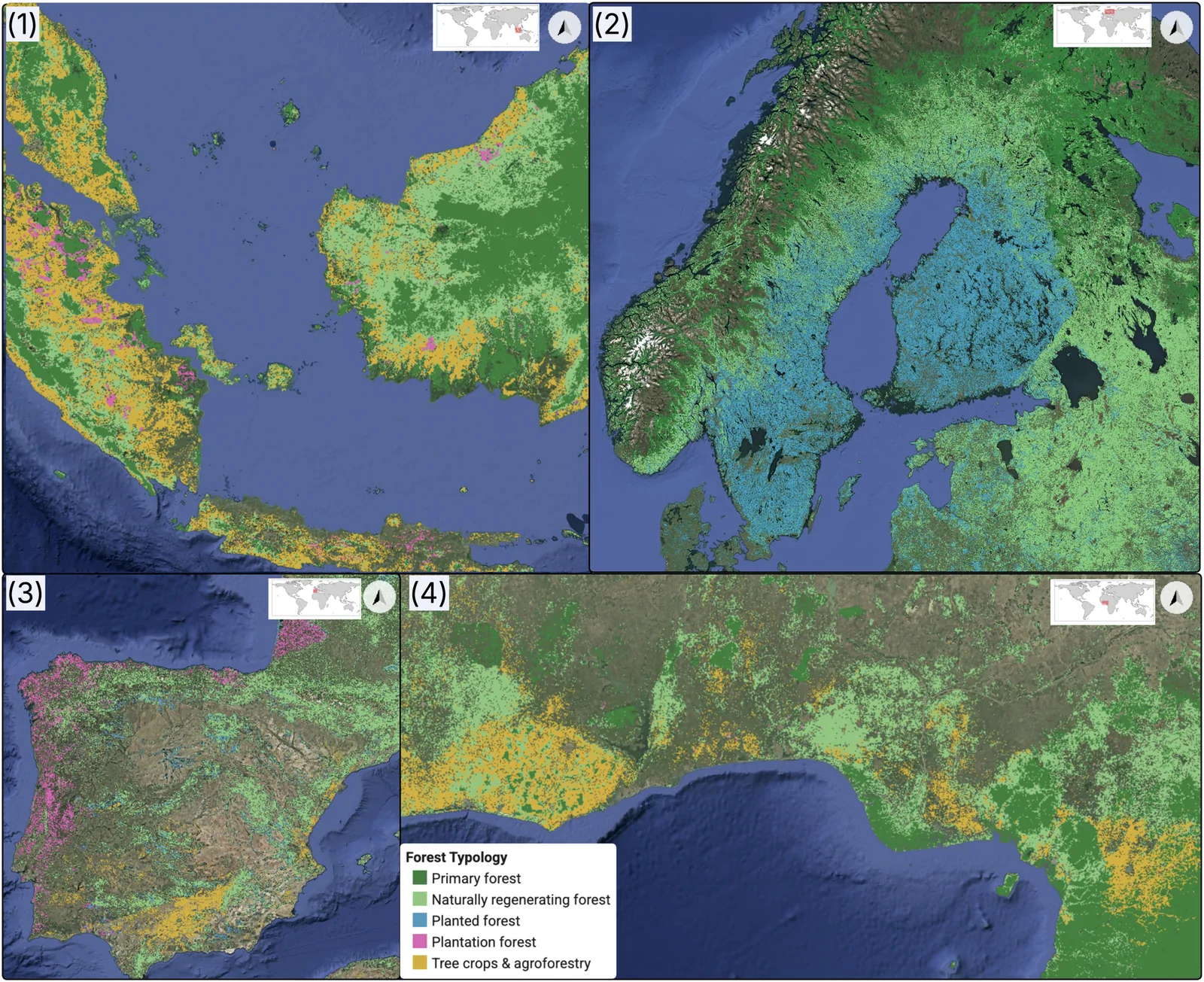
Regional hotspot examples: (1) extent of tree crops, plantations, and remaining natural forests in South-East Asia, (2) planted forests in Scandinavia, (3) plantation and planted forests and tree crops on the Iberian Peninsula and in southern France, (4) agroforestry systems in West Africa. Inset maps show the geographic location of each panel.

**Fig. 3:**
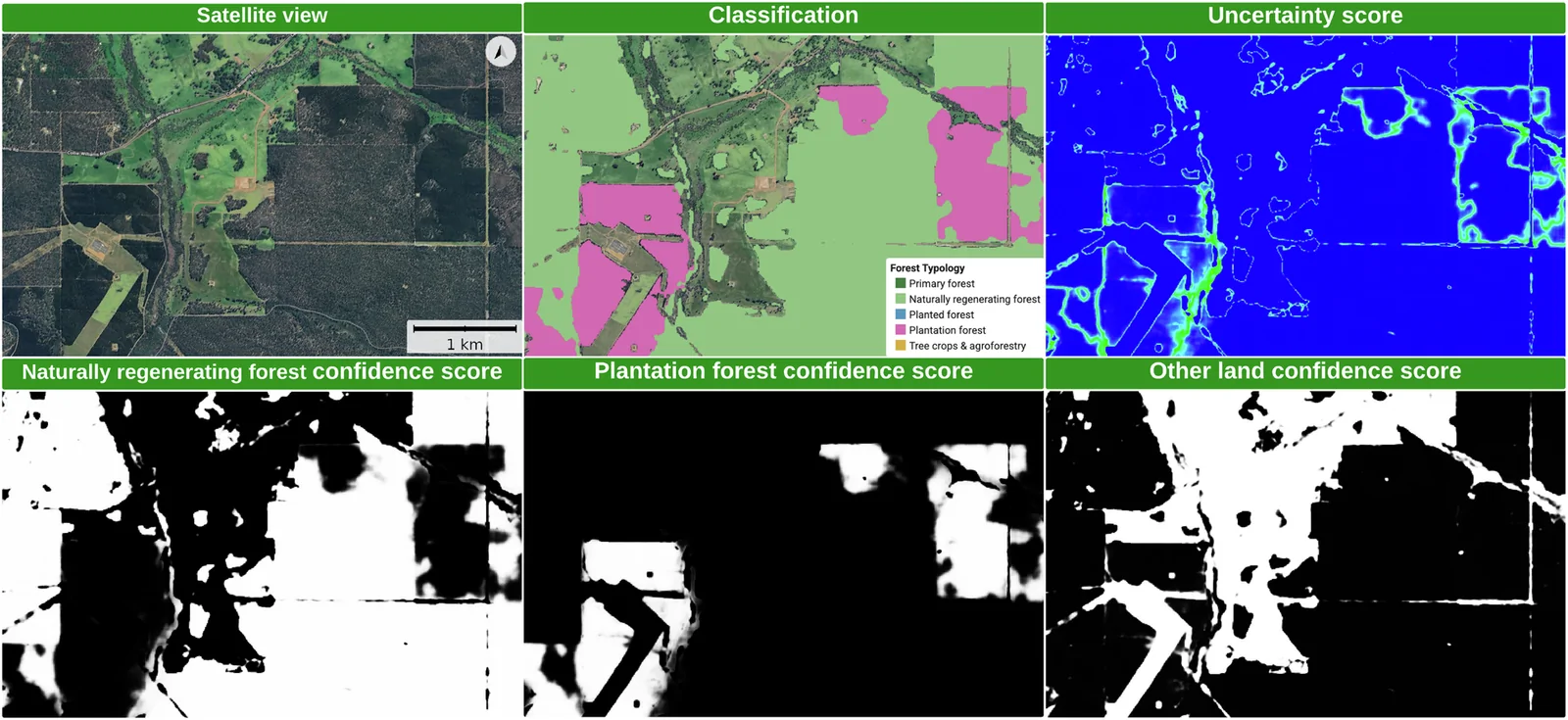
Zoomed-in example over natural and plantation forests in South-East Australia (extent per panel: 5 km × 3.5 km). From top left to bottom right: satellite imagery (© Google Earth, Maxar Technologies, Airbus); Forest Typology predictions; estimation uncertainty score; confidence score of Naturally Regenerating Forest; confidence score of Plantation Forest; confidence score of Other Land use.

This paper introduces the Forest Typology (ForTy) v1 dataset, a global 10 m resolution map for the year 2020, classifying all land into six categories aligned with FAO forest definitions, which are referenced by the EUDR and other forest accounting frameworks: (1) Primary forest, (2) Naturally Regenerating forest, (3) Planted forest, (4) Plantation forest, (5) Tree crops and Agroforestry, and (6) Other land. Rather than hard categorical labels, the dataset distributes per-class relative confidence scores, enabling users to adapt the classification to regional contexts and application-specific precision–recall trade-offs^23^. We describe the methodology, training data, and validation of a scalable deep learning pipeline that estimates forest type from Earth observation data. The resulting global map is shown in Fig. 1, with regional-scale examples in Fig. 2 and detailed zoom-ins in Fig. 3.

The following factors proved useful for training a single, globally consistent model to estimate forest type at any location on Earth:

1. **Three-stage cascaded training** at different label granularities, temporal extents, and dataset sizes, enabling the use of heterogeneous reference data sources;
2. **Geospatial satellite embeddings**, which encode multi-source, multi-temporal satellite observations into compact per-pixel representations;
3. **Label harmonization**, combining diverse regional and global reference datasets into a globally consistent training corpus with hierarchical label structures;
4. **Weakly supervised learning with partial labels**, allowing the model to learn from samples labeled at different levels of the class hierarchy; and
5. **Active learning**, iteratively refining training data in under-represented geographies and challenging biomes.

We validated the map against an independent, globally stratified random sample of 9,100 sites, of which 8,190 were fully resolved (at the time of writing this manuscript) through expert consensus and used for accuracy estimation. Two trained experts independently evaluated each site at 10 m resolution. The stratified overall accuracy across all six classes reaches 90.2% (SE = 0.3%). For the binary natural forest versus non-natural classification, overall accuracy reaches 94.8% (SE = 0.3%). All map data and training code are publicly available to support deforestation risk assessment, conservation planning, and forest monitoring research.

## Methods

**Fig. 4:**
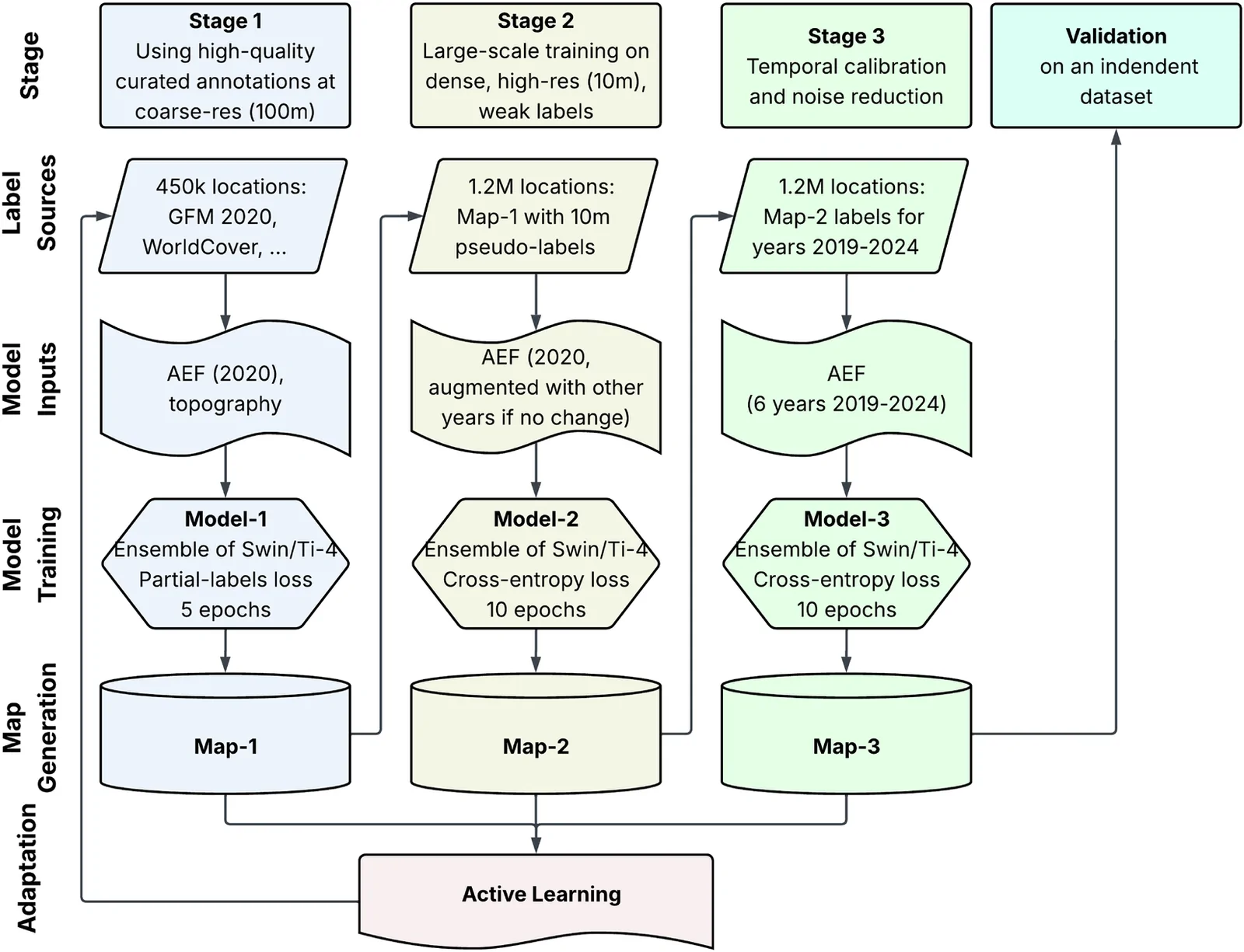
Overall study design diagram for the multi-stage data flow, model training, and map generation.

Figure 4 provides an overview of the study design, from training data curation through three-stage model training to global map generation and validation.

A key challenge is that the available high-quality reference data for forest types is limited, geographically uneven, and predominantly consists of point annotations denoting the majority class within a $\sim 100\,{\rm{m}}\times 100\,{\rm{m}}$  area—not dense segmentation masks at 10 m resolution. Moreover, the diversity of forest and tree crop land uses across the world’s biomes means that obtaining accurate training labels in every region is essential, yet practically infeasible at scale. To address these data constraints, we develop a cascaded three-stage design: the first stage trains on the available curated high-quality reference data—predominantly point-level annotations—to learn globally representative class patterns; the second stage uses these initial predictions as pseudo-labels, refined by ancillary high-resolution datasets, to scale training to over one million densely labeled patches at 10 m resolution across all land regions; and the third stage leverages multi-year temporal context to improve temporal consistency and reduce noise.

Each stage trains a model (Model-1, Model-2, Model-3) to perform dense semantic segmentation on 128×128 pixels at 10 m resolution (1,280 × 1,280 meter patches), classifying each pixel into one of six classes. Once trained, a model is applied for global inference, which can also cover years different from the training year, based on the input data years. Each successive stage beyond the first derives its training labels from the previous stage’s per-class probability maps, used as pseudo-labels and refined with ancillary data.

### Class definitions

We adopt a six-class forest typology that maps to EUDR regulatory requirements. Following the FAO Global Forest Resources Assessment^3^, the EUDR defines *forest* as land spanning more than 0.5 hectares with trees higher than 5 meters and a canopy cover of more than 10%, excluding land that is predominantly under agricultural or urban land use^8^. Within this forest definition, the six classes are distinguished by land use, management intensity, and rotation period as observed for the target year 2020.

- **Class 1: Primary Forest** (*Primary*). Natural forest of native species with no visible indications of human-caused disturbance. Primary forests include areas regenerating after natural disturbances such as wildfires, pest outbreaks, and wind damage, provided no human-caused land-use change is evident. Forests affected by low-intensity natural disturbances or fragmentation—where the canopy was never fully removed—may retain their classification as primary forest if no clearly visible indications of human activity are present.
- **Class 2: Naturally Regenerating Forest** (*NatReg*). Forests predominantly established through natural regeneration after human-caused disturbances. This class includes secondary forests recovering from disturbance and mixed forests where natural regeneration dominates. In practice, this class encompasses a heterogeneous set of successional stages and disturbance histories^29^: it includes post-agricultural recovery (abandoned cropland or pasture reverting to forest), post-clearcut regrowth, post-logging regrowth where the original canopy was substantially removed, and forests of mixed planted and natural origin where natural regeneration constitutes the majority of the growing stock. In cases when human-caused and natural disturbances interact and overlap, this class may include forest regenerating after natural disturbances, e.g. within burned areas adjacent to logging. The NatReg class thus captures forests where a human-caused stand-replacing disturbance or land-use conversion has occurred, followed by natural recovery. Because this class spans a wide range of regeneration ages—from young regrowth of a few years to mature secondary forests several decades old—areas within this class may differ substantially in structure, biomass, and biodiversity value.
- **Class 3: Planted Forest** (*Planted*). Forests established predominantly through planting or seeding. We introduce an operational 40-year rotation period threshold to distinguish the planted forest class from the more intensively managed plantation forest class defined below.
- **Class 4: Plantation Forest** (*Plantation*). Intensively managed forests characterized by one or two species, even age, regular spacing, and with a rotation period of ≤40 years.
- **Class 5: Tree Crops and Agroforestry** (*TreeCrop*). Agricultural plantations producing commodities other than wood (e.g., oil palm, rubber, fruit orchards), including agroforestry systems. Classified as agricultural land use under EUDR.
- **Class 6: Other Land** (*Other*). All non-tree land uses, sparse woody vegetation (canopy cover $\mathrm{ < 10 \% }$), other wooded land, trees in urban environments, and small tree patches that do not meet forest thresholds.

**Fig. 5:**
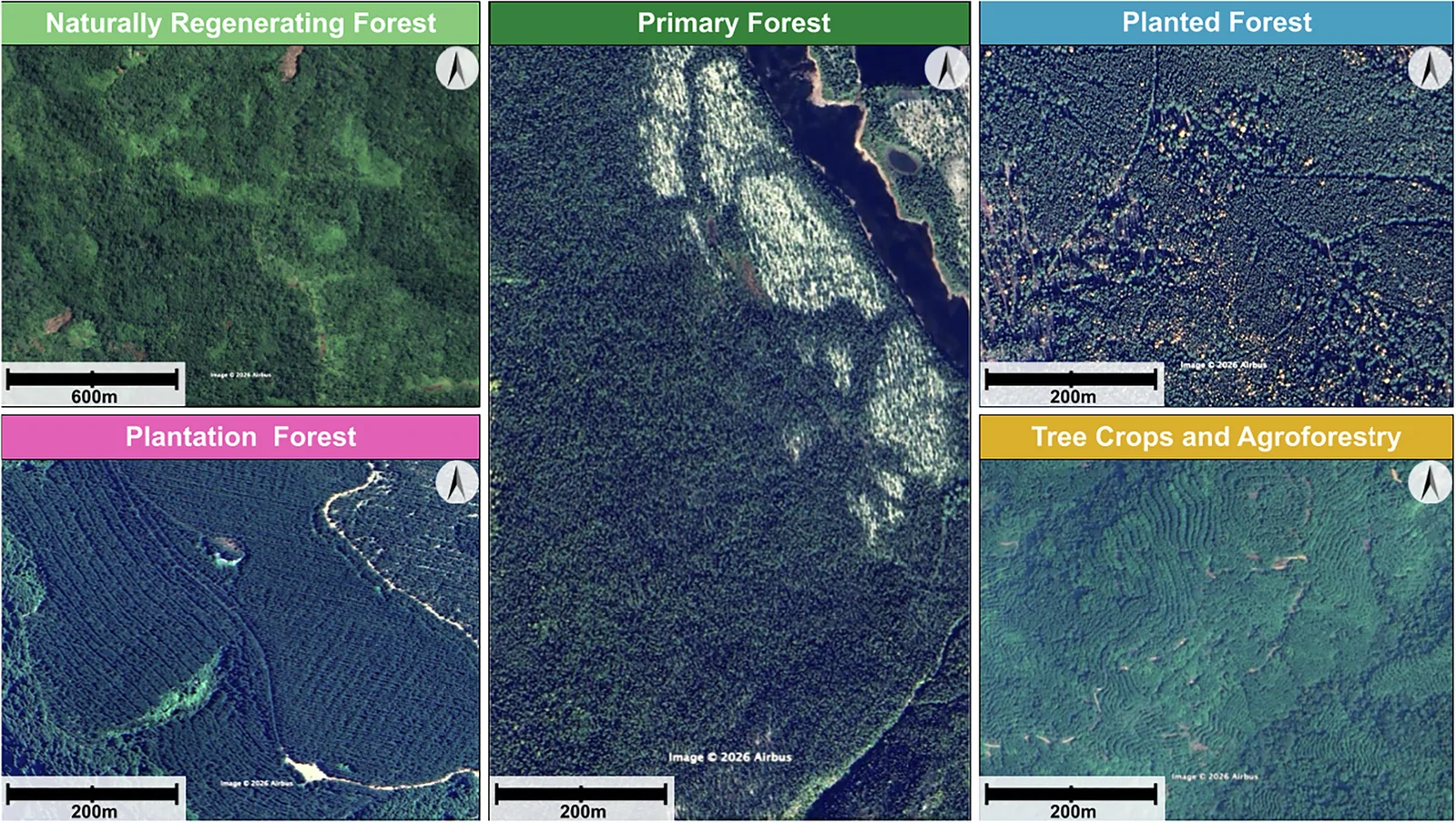
Examples of high-resolution satellite imagery for the five forest and tree cover classes: (1) Primary forest in boreal Quebec, Canada; (2) Naturally regenerating tropical forest in Papua New Guinea; (3) Planted forest in Germany; (4) *Pinus radiata* plantation in the Biobío region, Chile; (5) Rubber (*Hevea brasiliensis*) tree crop plantation in Xishuangbanna, Yunnan, China. Imagery © Google Earth, Maxar Technologies, Airbus.

Figure 5 shows examples of how the five forest and tree cover classes might appear in high-resolution satellite imagery.

### Planted vs. plantation forest: the 40-year rotation proxy

The FAO and EUDR definitions distinguish planted forest from plantation forest by management intensity criteria—monoculture, even-age class, and regular spacing—rather than by a specific rotation period. Because management intensity is not directly observable from satellite imagery, we adopt an operational 40-year rotation period threshold as a potentially observable proxy in time series data: planted forests with rotation periods exceeding 40 years are classified as planted forest, while those with shorter rotations are classified as plantation forest. This threshold was chosen to separate fast-rotation industrial plantations from slower-rotation planted forests. We acknowledge that this criterion is an operational simplification: it may not generalize across all biomes, silvicultural systems, or country-specific management traditions. In some regions, rotation periods near 40 years may not reliably distinguish management intensity, and planted forests with long rotations but intensive management (or vice versa) may be misclassified.

### Other wooded land (OWL)

The EUDR recognizes other wooded land—areas with 5–10% canopy cover, or combined tree, shrub, and bush cover exceeding 10%—as a distinct category. Conversion of primary forest to OWL constitutes forest degradation under the regulation. In the Forest Typology map, OWL is subsumed into the *Other* class and is not resolved as a separate class. This means that a transition from forest to OWL cannot be directly detected as a within-class change in the Forest Typology map; it would instead appear as a transition from a forest class to Other. Users interested in monitoring forest-to-OWL transitions—which may constitute degradation under the EUDR—should combine the Forest Typology baseline with canopy cover change products that can resolve the 10% canopy cover threshold. Separating OWL as a distinct class is a target for future versions of the dataset.

### Input data

The primary input to all model stages consists of AlphaEarth Foundations (AEF) embeddings^30^. AEF compresses multi-source, multi-temporal satellite observations (Sentinel-1, Sentinel-2, Landsat, ALOS PALSAR-2, and others) into annual per-pixel embedding vectors at 10 m resolution. Each embedding encodes spectral, temporal, and contextual information for a given pixel-year. For Stage 1 (Model-1), we supplement the AEF embeddings with topographic features derived from the Copernicus DEM at 30 m, upsampled to 10 m^31^. These features comprise elevation above sea level, slope, and slope aspect. Stages 2 and 3 use AEF embeddings as the sole input modality, covering 2019–2021 and 2019–2024, respectively.

### Training data

We construct training data for the three model stages from a combination of curated reference datasets, external regional datasets, active learning campaigns, and pseudo-label generation from preceding stages. Table 1 summarizes all training data sources.

**Table 1:** Training label data sources (Model-1: 450,275 samples; Model-2 uses the 1.2M pseudo-labeled patches and a subset of Model-1 sources; Model-3 uses only the 1.2M patch locations).

Source	Samples	Description
*Core reference datasets:*		
GFM 2020	262,837	Crowd-sourced global forest management labels^18^ mapped to six-class Forest Typology scheme. The annotations were first updated for 2020 by IIASA, and subsequently refined by Google through distributed human review, AI-assisted label processing, and rule-based heuristics.
ESA WorldCover v3	96,000	Augments GFM 2020 data with negative non-tree land cover classes (grassland, built-up, bare, ice, water, wetland, cropland) that provide “other” labels. Pixels with canopy height ≥1 m are masked to *unknown*^32^.
*External tree crop and plantation datasets:*		
CIAT Vietnam	12,056	Tree crop labels from CIAT/CGIAR in Vietnam^58^.
Wang *et al*. rubber	3,887	Rubber plantation training samples across Southeast Asia and Africa^59^.
CIAT Ghana	3,443	Tree crop labels from CIAT/CGIAR in Ghana^58^.
LACUNA tree crops	2,732	Tree crop labels for Ghana from the CERSGIS/Lacuna Fund^60^.
SERVIR Mekong	2,228	Rubber and other plantation labels across the Mekong region^61^.
Descals oil palm	972	Oil palm planting year labels^62^.
Fricker Ucayali palm	933	Oil palm polygons in Ucayali, Peru^63^.
Sheil *et al*. correction	481	Rubber deforestation correction labels^64^.
Descals coconut	327	Closed-canopy coconut palm map^65^.
Becerra cacao Peru	213	Cacao plantation polygons in Amazonia Peruana^66^.
Jin cashew Benin	212	Smallholder cashew plantations in Benin^67^.
*Internal annotations:*		
Internal annotations	19,911	Google-internal annotation campaigns: point and polygon annotations for tree crops, forests, and other land uses.
*Active learning (AL)*		
AL Round 1: IFL-based	11,842	Primary/NatReg confusion correction using Intact Forest Landscape boundaries^14,39^.
AL Round 1: CORINE-based	1,505	Tree crop precision/recall corrections using CORINE Land Cover 2018^68^.
AL Round 1: model-based	8,784	Tree crop corrections from model disagreement, map inspection, and precision loss analysis.
AL Round 2: SDPT-based	1,016	Planted/plantation corrections in China, USA, Japan, Chile, S. Korea using SDPT^16^.
AL Round 3: expert review	19,880	Expert-verified corrections: vineyard/banana overclassification, Miskito pine, missing plantations, primary forest overestimation^39^.
*Stage 2 pseudo-labels (Model-2 and Model-3 only):*		
1.2M global patches	1,200,000	Globally distributed random locations. Labels from Model-1 pseudo-labels refined by ancillary datasets (Table 2).

### Model-1 label construction

The preprocessing pipeline constructs Model-1 labels by combining and filtering three source categories:

- **GFM reference data**^18^: At the core, we rely on expert labels from the original Global Forest Management (GFM) dataset that are mapped to the six-class Forest Typology scheme for the year 2020, as described below.
- **WorldCover**^32^: Non-tree land cover classes (built-up, bare, water, ice, grassland, wetland, moss/lichen, cropland) are assigned to “Other” (class 6). Tree cover, cropland, and mangrove classes are masked to *unknown* to preserve label ambiguity if not already annotated via GFM.
- **Supporting and active-learning datasets**: Multiple regional and global reference sources (Table 1) are mapped into the six-class label space. Some sources provide partial labels—multiple annotations representing the set of possible classes. Unlabeled pixels are set to *unknown* and excluded from the loss.

### GFM 2020 training data adaptation

IIASA labeling team initially updated training labels in GFM 2015 to 2020. We additionally adjust these labels toward EUDR class definitions using a combination of automated reclassification and human review. We use non-expert annotators and Gemini-assisted high-resolution imagery analysis to identify and resolve inconsistencies between original source labels and EUDR definitions for planted and plantation samples. GFM class 53 (agroforestry and other ambiguous tree cover) is resolved using a heuristic that considers canopy height^33^, building proximity^34^, WorldCover^32^ tree coverage, and country of origin. A canopy height filter reclassifies forest pixels (classes 1–4) to “other” when the canopy height model reports height below 1 m.

### Model-2 label construction

Training labels for the 1.2M global random patches fuse the Model-1 per-class probability map with ancillary geospatial datasets (Table 2) through a hierarchical assignment procedure:

1. **Natural forest identification**. The Natural Forests of the World (NFW) 2020 map^23^ provides an independent naturalness estimate. This layer is cross-referenced with Model-1 forest probabilities: pixels with NFW scores $\,\ge \,0.5$ are classified as primary or naturally regenerating forest based on the dominant Model-1 class.
2. **Managed forest and tree crop assignment**. For pixels classified as tree cover by WorldCover^32^, the dominant Model-1 class (planted, plantation, or tree crops) is assigned when its probability exceeds 0.5.
3. **Non-forest assignment**. WorldCover non-tree classes are assigned to “other”. High-resolution building and road data^34,35^ override forest classifications. Pixels with Model-1 “other” probability $\,\ge \,0.94$ are classified as “other”.
4. **Regional external data corrections**.

- SDPT^16^ data from Canada is used for additional planted forest annotations in Canada.
- MapBiomas Collection 10 *Deforestation and Secondary Vegetation* data^36^ is used in Brazil to refine the Primary–NatReg distinction. We use the trajectory classification which encodes land-use history over the full 1985–2020 MapBiomas time series to re-adjust labels in two ways: (a) pixels classified as primary forest by Model-1 are relabeled to naturally regenerating forest where MapBiomas reports secondary vegetation (value 3), reflecting that the site has undergone at least one anthropic land-use episode and the current forest cover represents regrowth; (b) pixels classified by Model-1 as planted forest, plantation, or tree crops are relabeled to naturally regenerating forest where MapBiomas reports primary vegetation (value 2), correcting cases where Model-1 misclassified stable natural forest as a managed type.
- USDA cropland data^37^ is used over the United States to override planted/plantation classifications in favor of tree crops for known agricultural tree crops.

5. **GFC temporal adjustment**. Global Forest Change (GFC)^7^ tree cover loss year is used to reclassify pixels with recent tree cover loss to the “other” land use class. The model may still predict a forest or tree crop class where strong signs of regrowth or planting are present. Wildfires are excluded from this reclassification using forest loss driver data^38^.

**Table 2:** Ancillary datasets used for Model-2 pseudo-label refinement.

Ancillary Source	Role in Model-2 Label Construction
ESA WorldCover 2020^32^	Land cover class filtering for forest vs. non-forest assignment.
Natural Forests of the World 2020^23^	Independent naturalness estimate for *Primary*/*NatReg* distinction.
Meta Canopy Height^33^	Height-based label filtering (forest classes require ≥1 m).
Google Buildings & Roads^34,35^	Built area detection overrides forest to *Other* class.
Hansen GFC 2025^7^	Year-specific deforestation detection.
MapBiomas^36^	*Primary* → *NatReg*; and *Planted* → *NatReg* (Brazil).
USDA Cropland Data Layer^37^	*TreeCrop* identification in the United States.
SDPT^16^	Selected planted forest detections.
Wildfire driver data^38^	Wildfire exclusion from Hansen loss reclassification.

### Model-3 label construction

Multi-temporal training labels for Model-3 are generated using a heuristic based on the predictions of six inference maps from Model-2 (Stage 2), one per year from 2019 to 2024. For each pixel:

- If no tree cover loss was detected according to GFC 2025 data^7^ and the estimated probability of the *Other* class changes by less than 0.7 across years (absolute difference between per-year probabilities), the class with the highest *mean probability* is used—providing noise reduction through averaging (note that there might still be change, within the *Other* or *TreeCrop* classes, but it does not affect our taxonomy).
- Otherwise—if tree cover loss was detected, or if the absolute change in *Other* class probability across years exceeds 0.7—*individual per-year predictions* are preserved, allowing the model to learn genuine land-cover transitions.

Across all stages, refinements and corrections apply only to the training labels; no post-processing is applied to the model predictions when generating the final map.

### Active learning

We employ an active learning workflow to improve classification quality in under-represented geographies and for classes that are difficult to distinguish globally. The process follows a cyclical *model training* → *inference* → *map generation* → *expert feedback* → *location identification* → *annotation* → *retraining* loop.

After each training iteration, we generate a global inference map, which domain experts inspect to identify systematic misclassifications. These inspections—triggered primarily by expert feedback and visual map assessment (including historical analysis)—reveal regions and land-use types with insufficient training data coverage. For each identified error pattern, we create a targeted annotation campaign: annotators visually interpret high-resolution imagery, consult reference products, and review time series data at flagged locations, assigning corrected labels following the six-class Forest Typology scheme.

We conducted three major rounds of active learning, contributing approximately 43,000 additional training samples in total. All active learning samples are merged with the original training data and treated identically during training.

1. **Round 1** (22,131 samples): The first campaign targeted three main error patterns identified through initial map inspection: (a) areas of confusion between naturally regenerating and primary forests, where expert-verified Intact Forest Landscape (IFL) labels^14,39^ resolved ambiguity (11,842 samples); (b) regions with significant precision or recall losses for tree crops, particularly in Southeast Asia, West Africa, and South America (7,254 samples); and (c) areas with potential under-prediction of planted and plantation forests in China (407 samples).
2. **Round 2** (1,016 samples): The second campaign focused on both precision- and recall-motivated corrections in regions with a large fraction of planted and plantation forests: China, northwestern and southeastern USA, Japan, Chile, and South Korea.
3. **Round 3** (19,880 samples): The third campaign addressed a broader set of systematic errors: (a) over-classification of tree crops, including vineyards in Europe and banana plantations in West Bengal, India; (b) misclassification of natural pine forests as planted within the Miskito pine forests ecoregion in Central America; (c) primary forest overestimation in areas with noticeable human impact (Canada, Siberia, Turkey, USA); and (d) missing oil palm plantations in Africa and Indonesia and missing tropical plantations more broadly.

### Model architecture and training stages

All three stages use the Swin Transformer v2 architecture^40^, a common hierarchical vision transformer that computes representations through shifted windows of self-attention^41^. We use the *Tiny* variant with patch size 4 (*SwinV2-Ti/4*) and a dense segmentation head. At each stage we train an ensemble of five independently initialized SwinV2-Ti/4 models, each producing separate predictions. During inference, predictions are averaged across ensemble members to improve model reliability.

All stages share the optimizer configuration listed in Table 3. Stage-specific parameters are noted in Table 4.

**Table 3:** Shared training configuration across all stages.

Parameter	Value
Optimizer	Adam
Learning rate	0.001
Weight decay	0.00003
LR schedule	Cosine decay with 10% warmup
Gradient clipping	Max norm 1.0
Batch size	512
Train data augmentation	Random spatial roll, flip, and rotation

**Table 4:** Stage-specific training parameters.

Parameter	Model-1	Model-2	Model-3
Epochs	5	10	10
Input modalities	AEF + elevation	AEF	AEF
Input years	2020	2020 (2019, 2021)	2019–2024
Temporal sampling	2020	2020 (or random year if no change)	All 6 jointly
Loss function	Partial label	Softmax CE	Softmax CE
Output classes	6	6	6 for each year

### Stage 1: Training on curated reference data

Model-1 trains a base classifier on 450,275 expert-labeled and curated reference samples (Table 1). The input consists of AEF embeddings for the year 2020 and elevation features.

Model-1 uses a *partial label loss* with an entropy regularization weight of 0.1^42,43^. Fully labeled examples contribute standard cross-entropy loss. Partially labeled examples enable the model to learn from heterogeneous annotation granularity by allowing ambiguous, but limited, outputs.

### Stage 2: Large-scale training

Model-2 scales training to over 1.2 million globally distributed, densely annotated samples at 10 m resolution ($\mathrm{1,280}{\rm{m}}\times \mathrm{1,280}{\rm{m}}$ patches). The input consists of AEF embeddings.

Additionally, Model-2 applies temporal augmentation by randomly selecting one of three input years (2019, 2020, or 2021) per training example when there is no change between years according to Global Forest Change (GFC)^7^. This increases the effective training set diversity by exposing the model to inter-annual variability in vegetation phenology and satellite acquisition conditions.

Model-2 uses the standard softmax cross-entropy loss (not partial labels) and trains for 10 epochs. The trained Model-2 ensemble is then applied independently to each year from 2019 to 2024, producing per-class probability maps that serve as input to Stage 3.

### Stage 3: Temporal calibration

The third stage performs temporal calibration by jointly training on 1,200,000 samples across six years (2019–2024). This stage improves temporal consistency of the 2020 prediction by leveraging context from neighboring years while preserving land cover changes. For this, the model sees inputs for all 6 years at the same time, and has to make predictions for each year.

All six years (2019–2024) of AEF composites are concatenated along the temporal axis, producing a six-frame input tensor per training example. The temporal patch size for the transformer inputs is set to 6, encoding the full six-year sequence at each spatial location within a single patch token. Cross-year information flow occurs through the spatial encoder and decoder.

Similarly to Model-2, Model-3 uses softmax cross-entropy loss and trains for 10 epochs. For the final 2020 map, the year-2020 slice of the ensemble output is extracted.

### Map construction

We create an inference dataset covering all land areas at each stage using the same procedure. The final model (or ensemble of models) estimates class probabilities for each inference sample. To reduce tiling artifacts, we perform inference with overlapping samples: adjacent sample centers are spaced 210 m apart, while each sample spans $\mathrm{1,280}\,{\rm{m}}\times \mathrm{1,280}\,{\rm{m}}$. Estimates for overlapping pixels are weight-averaged using the inverse Euclidean distance from each pixel to its respective sample center. This overlapping inference strategy produces a seamless final map.

### Class probabilities and uncertainty

The per-class pseudo probabilities (or relative confidence scores) are computed as the arithmetic mean of softmax outputs from five independently initialized models, a standard ensemble strategy that reduces epistemic uncertainty and improves calibration^44,45^. The overlapping inference strategy provides additional smoothing. Aleatoric uncertainty—intrinsic ambiguity from overlap between forest types—is not explicitly modeled but is partially captured by the probability distribution itself: pixels near class boundaries naturally receive less confident predictions. The probabilities have not been post-hoc calibrated, so they should be interpreted as relative confidence scores rather than calibrated posterior probabilities^23,46^.

## Data Records

The dataset is available at Figshare^47^. It is also available on Google Earth Engine (GEE) under asset ID projects/nature-trace/assets/forest_typology/forest_typology_2020_v1_0_collection

The dataset is licensed under the Creative Commons Attribution 4.0 International License (CC-BY 4.0). It contains 613 zip archives, one for each UTM cell that contains land. The zip files are named raster_{LON_ZONE}{LAT_ZONE}.zip, where LON_ZONE is a number between 1 and 60, and LAT_ZONE is a capital letter for the latitudinal zone. Each archive contains multiple smaller Cloud Optimized GeoTIFF (COG) raster files.

The Forest Typology 2020 v1 dataset consists of a global map of per-class relative confidence scores at 10 m resolution covering all land areas between 65 S^°^ and 84 N^°^ latitude. The map uses the Universal Transverse Mercator (UTM) coordinate system, has a spatial resolution of 10 m per pixel, and contains 8-bit unsigned integer (*uint8*) values (0–250) representing quantized probability scores.

The primary data record is a five-band raster:

**Table:** 

Band	Band name	Type	Min	Max
1	PrimaryForest	*uint8*	0	250
2	NaturallyRegeneratingForest	*uint8*	0	250
3	PlantedForest	*uint8*	0	250
4	PlantationForest	*uint8*	0	250
5	TreeCropsAndAgroforestry	*uint8*	0	250

The relative confidence score for the sixth class (*Other*) is obtained as the complement: ${p}_{{\rm{other}}}\mathrm{=250}-{\sum }_{i\mathrm{=1}}^{5}{p}_{i}$. A categorical map is derived by assigning each pixel to the class with the highest confidence score (argmax). The relative confidence scores representation enables users to apply custom thresholds, assess estimation uncertainty, and compute uncertainty estimates for downstream applications.

### Classification layer dataset

For convenience, we release the extracted classification layer as a separate dataset on Figshare^48^. It is also available on Google Earth Engine under asset ID projects/nature-trace/assets/forest_typology/forest_typology_2020_v1_0_classification

It is a single-band raster collection in COGs, with a spatial resolution of 10 m per pixel, with values between 1 and 6 for the six classes of Forest Typology. The dataset is licensed under the Creative Commons Attribution 4.0 International License (CC-BY 4.0).

### Validation dataset

The *Global Validation of Forest Types* (GVFT) dataset (v0.6) is available at Figshare^49^ as a CSV file, licensed under the Creative Commons Attribution 4.0 International License (CC-BY 4.0). It contains 9,100 sample sites with geographic coordinates, sampling stratum, per-expert annotations, the consensus label, and the corresponding Forest Typology v1 map confidence scores for all six classes. Of these, 8,190 sites have been resolved through expert consensus; the remaining sites include 738 samples pending consensus and 172 samples marked as uncertain.

## Technical Validation

### Global validation of forest types dataset

**Fig. 6:**
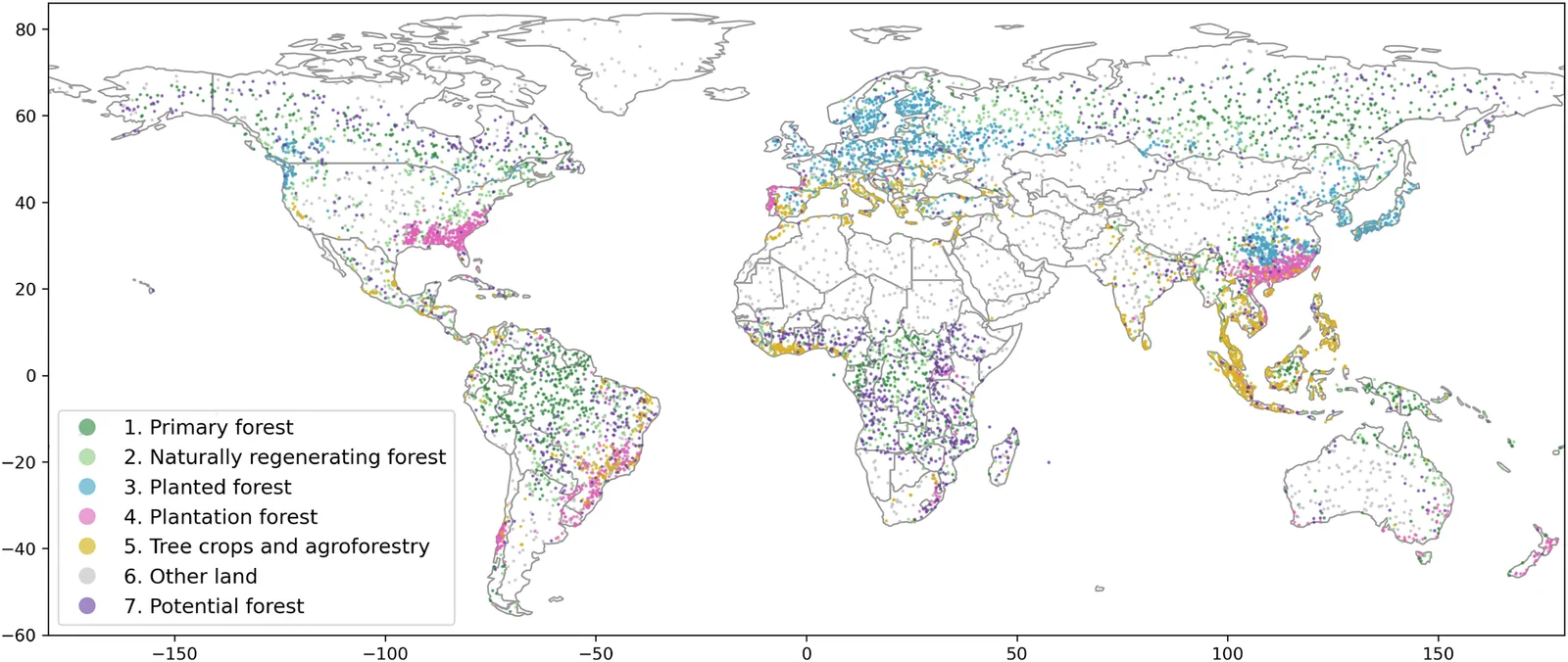
Sample locations and strata of the GVFT dataset.

We conducted a global validation campaign using IIASA’s Geo-Wiki platform^50^. A global stratified random sampling design was adopted, with seven preliminary map-based strata to ensure coverage of rare classes: the six Forest Typology classes plus a Potential Forest stratum, which captures areas mapped as tree cover by external layers such as Hansen/GLAD^7^ and ESA WorldCover^32^ but not assigned to any of the five forest and tree cover classes. We randomly distributed 1,300 sample sites per stratum (9,100 in total). Table 5 summarizes the stratum areas and sample counts, and Fig. 6 visualizes the spatial distribution of sites across strata.

**Table 5:** Validation strata: map-based area, total sample count, and breakdown of excluded samples.

Stratum	Area (Mha)	Samples	Unresolved	Not sure
1. Primary Forest	2,123.7	1,300	126	9
2. Naturally Regenerating Forest	1,797.9	1,300	133	18
3. Planted Forest	204.7	1,300	146	38
4. Plantation Forest	118.0	1,300	163	40
5. Tree Crops and Agroforestry	145.8	1,300	83	44
6. Other Land	7,644.6	1,300	12	1
7. Potential Forest	1,339.2	1,300	75	22
**Total**	**13,374**	**9,100**	**738**	**172**

Unresolved sites had not yet completed expert consensus; “not sure” sites were labeled as uncertain by at least one expert.

Of the 9,100 sites, 8,190 were fully resolved through expert consensus and constitute the Global Validation of Forest Types (GVFT) dataset used for all accuracy reporting. Of the remaining sites, 172 (1.9%) were labeled “not sure” by at least one expert due to insufficient satellite imagery or inherent land-use ambiguity; the rest had not yet been fully resolved in consensus meetings at the time of analysis. This sample size supports accuracy estimation at continental and regional levels.

Two trained experts independently evaluated each sample site at two spatial scales: a $30\,{\rm{m}}\times 30\,{\rm{m}}$ context window (3×3 grid of 10 m pixels) and the central $10\,{\rm{m}}\times 10\,{\rm{m}}$ pixel. For simplicity, all accuracy results reported in this paper are based on the central 10×10 m pixel. Experts based their assessments on the visual analysis of very high resolution imagery, contextual geospatial layers, long-term Landsat, Sentinel-2, and Sentinel-1 time series, and their regional expertise. For challenging cases, experts additionally consulted ground-truth information and area specialists. Disagreements were resolved through consensus meetings.

As shown in Table 5, the 172 “not sure” labels and 738 unresolved sites are not uniformly distributed across classes. The “not sure" responses are concentrated in the managed and transitional strata—Tree Crops (44), Plantation (40), and Planted Forest (38)—whereas Primary Forest (9) and Other Land (1) have very few. Similarly, unresolved sites are most frequent in Plantation (163) and Planted Forest (146) strata. This pattern indicates that expert uncertainty is highest where the distinction between forest types depends on management history and land-use intent rather than on observable canopy properties—precisely the class boundaries that are most challenging for remote sensing. Excluding these sites from accuracy estimation may therefore yield slightly biased accuracy estimates.

### Validation annotation protocol

The Global Validation of Forest Types (GVFT) dataset uses 13 annotation labels, grouped into three categories. Expert annotators select from these labels when interpreting each sample site. The mapping of GVFT labels to Forest Typology classes is outlined in Table 6.

**Table 6:** Forest Typology Class Definitions and Aggregation Schema for Validation Labels.

Forest Typology Class	Operational Definition	Validation Labels (GVFT)
**Class 1: Primary Forest**	Naturally regenerating forest of native species with no clearly visible indications of human activity and undisturbed ecological processes. Area $\, > \,0.5{\rm{ha}}$.	• Primary forest • Burnt primary forest
**Class 2: Naturally Regenerating Forest**	Forests predominantly established through natural regeneration. Includes secondary recovery, native/introduced mixed regeneration, or coppice. Width >20m if shelterbelt.	• Naturally regenerating forest
**Class 3: Planted Forest**	Forests predominantly established through planting or seeding where the rotation period $\mathrm{ > 40}{\rm{years}}$.	• Planted forest
**Class 4: Plantation Forest**	Intensively managed, even-aged monocultures with regular spacing. Rotation period $\le 40{\rm{years}}$. Planted Teak is always grouped here.	• Plantation forest
**Class 5: Tree Crops and Agroforestry**	Agricultural plantations producing commodities other than wood (e.g., rubber, oil palm, fruit orchards, shaded cocoa/coffee). Treated as agricultural land under EUDR.	• Tree crops • Agroforestry (mixed) • Agroforestry (nature-close)
**Class 6: Other Land**	Residual non-tree land uses, sparse woody vegetation (canopy cover $\mathrm{ < 10 \% }$), or elements falling outside FAO forest specifications.	• Other land • Other wooded land • Other trees • Woodland • Silvopasture

Forest and tree-based annotation labels:

1. **Primary forest**. Naturally regenerating forest of native tree species with no clearly visible indications of human activities and undisturbed ecological processes. Minimum area $> 0.5\,{\rm{ha}}$.
2. **Naturally regenerating forest**. Forest predominantly composed of native or introduced trees established through natural regeneration following human-caused disturbances or over abandoned land use (agriculture, mining, etc.), including mixed stands where natural trees will dominate at maturity. Minimum area $> 0.5\,{\rm{ha}}$.
3. **Planted forest**. Forest predominantly established through planting or deliberate seeding (exceeding 50% of growing stock at maturity) with a rotation period exceeding $40\,{\rm{years}}$. Minimum area $> 0.5\,{\rm{ha}}$.
4. **Plantation forest**. Intensively managed planted forest meeting criteria of one or two species, even age class, and regular spacing. Rotation period is a maximum of $40{\rm{years}}$ (commercial teak is automatically classified here). Minimum area $> 0.5\,{\rm{ha}}$.
5. **Burnt primary forest**. Primary forest that has been significantly damaged by fire, where some trees are still standing (dead or alive).
  Agricultural and agroforestry annotation labels:
6. **Tree crops**. Clearly visible large- or medium-scale plantations where the main commodities are tree crops (e.g., rubber, oil palm) or orchards (e.g., olive, fruit trees).
7. **Agroforestry (mixed tree crops)**. Systems featuring cocoa, coffee, or shaded tree crops grown alongside other crops underneath (e.g., alley cropping, mixed tree crops, palms).
8. **Agroforestry (nature-close)**. Systems where tree crops are mixed with native trees, shrubs, or woody plantations.
9. **Silvopasture**. The practice of integrating trees, forage, and the grazing of domesticated animals in a mutually beneficial way.
  Other annotation labels:
10. **Other wooded land (Woodland)**. Land that is not technically a forest (canopy cover of 5% to 10%, or combined tree/shrub/bush cover >10%) exceeding $0.5{\rm{ha}}$. Currently falls outside the regulatory scope of the EUDR.
11. **Other trees**. Isolated elements falling outside standard forest thresholds, such as single trees on agricultural fields, pastures, tree shelterbelts, trees in urban/built-up areas, or small parks $< 0.5\,{\rm{ha}}$.
12. **Other land**. Areas containing no trees, but which can include shrubs.
13. **Not sure**. Used when there are insufficient data or imagery to decide on the correct class.

### Accuracy assessment

We evaluate the Forest Typology v1 map against the GVFT dataset using the central 10 m pixel of each sample site for the 2020 annotations. We report stratified Overall Accuracy (OA), User’s Accuracy (UA; precision), Producer’s Accuracy (PA; recall), and their respective standard errors (SE), following established best practices for accuracy assessment of land cover products^51,52^. We also report the F1-score, which is the harmonic mean of UA and PA:

$$F1=\frac{2\cdot {\rm{UA}}\cdot {\rm{PA}}}{{\rm{UA}}+{\rm{PA}}}$$

**Fig. 7:**
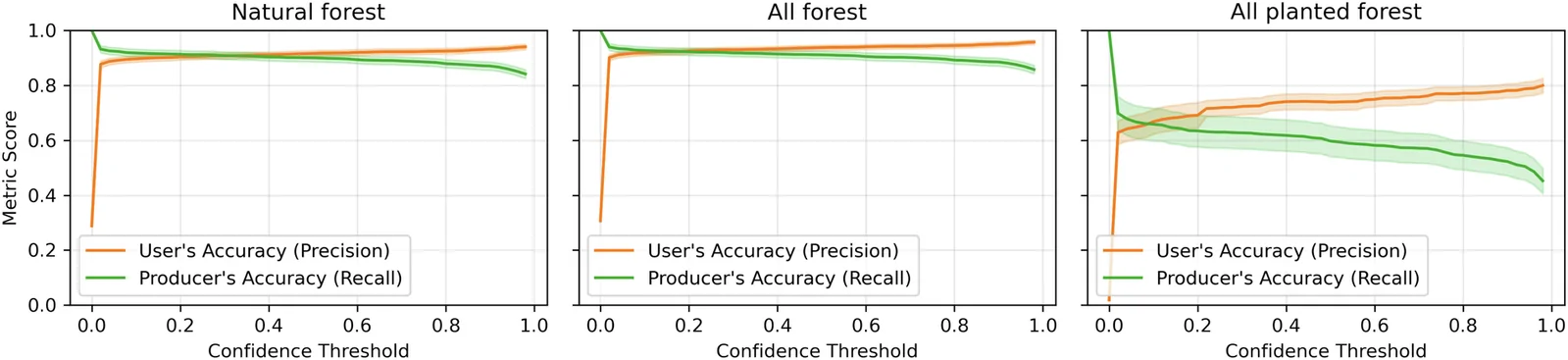
User’s accuracy (precision) and producer’s accuracy (recall) as a function of confidence threshold for the binary aggregations: (**a**) natural forest, (**b**) forest, and (**c**) planted forest. User’s accuracy measures the proportion of mapped pixels that are correct (i.e., the reliability of the map label), while producer’s accuracy measures the proportion of actual class instances that are detected. The shaded areas indicate 95% confidence intervals ($\pm 1.96\times SE$).

Because the Forest Typology map is distributed as a relative confidence scores raster, users can derive categorical maps at application-specific operating points by varying the class assignment threshold. The accuracy metrics reported below correspond to the default argmax rule (each pixel is assigned to the class with the highest confidence score). Users who require higher certainty may raise the confidence threshold for a target class, trading Producer’s Accuracy for User’s Accuracy. Figure 7 illustrates this trade-off by plotting UA and PA as a function of the confidence threshold for binary merged *natural* forest (primary + naturally regenerating), *all forests* (primary + naturally regenerating + planted + plantation), and *all planted forest* (planted + plantation) classes. The plots show 95% confidence intervals as 1.96× SE. We discuss threshold selection further in the Usage Notes.

We present accuracy at multiple levels of class aggregation to characterize the map’s performance across use cases of varying thematic granularity. Table 7 reports per-class accuracy for the full forest typology six-class scheme. The map achieves an overall accuracy of 90.2% (SE = 0.3%). Primary forest reaches the highest forest-class F1-score (82.9%), followed by naturally regenerating forest (72.6%) and plantation forest (66.7%). The planted forest class shows the lowest F1-score (58.2%), reflecting the difficulty of distinguishing planted forests from both naturally regenerating forests and plantations based on satellite observations alone. The “other” class achieves the highest overall performance (F1 = 96.4%). Because the “other” class dominates the stratified area weights, the high overall accuracy is substantially influenced by this class; the users should pay attention to the per-class F1-scores for the classes or class combinations that matter for their applications.

**Table 7:** Per-class accuracy for the full six-class scheme (consensus samples). OA = 90.22%, SE = 0.34%.

Class	F1	UA ± SE	PA ± SE
Primary	82.9%	77.9% ± 1.1%	88.5% ± 1.1%
NatReg	72.6%	81.1% ± 1.2%	65.8% ± 1.4%
Planted	58.2%	65.1% ± 1.8%	52.6% ± 3.7%
Plantation	66.7%	74.4% ± 3.1%	60.4% ± 3.2%
TreeCrop	62.1%	76.9% ± 2.8%	52.1% ± 4.2%
Other	96.4%	95.4% ± 0.4%	97.3% ± 0.2%

When primary and naturally regenerating forests are merged into a single *natural* forest class, and planted and plantation forests are merged into *all planted* forest (Table 8), overall accuracy increases to 93.9% (SE = 0.3%). For the binary classification of *natural* forest versus all other land cover, overall accuracy reaches 94.8% (SE = 0.3%). For the binary classification of *all forests* (classes 1–4) versus non-forest, overall accuracy reaches 95.5% (SE = 0.3%), with forest F1 = 92.6%. When merging *Planted* and *Plantation* forest classes, and the TreeCrop class into *Other*^21^, the remaining four-class scheme (Primary, NatReg, Planted, and Other) achieves OA = 90.9% (SE = 0.3%).

**Table 8:** Per-class accuracy with merged *all natural* (classes 1 and 2), *all planted* (classes 3 and 4), and *all forest* classes (classes 1, 2, 3, and 4).

Class	F1	UA ± SE	PA ± SE
All Natural Forests	90.9%	91.6% ± 0.5%	90.2% ± 0.7%
All Planted Forests	66.1%	74.3% ± 1.4%	59.5% ± 2.8%
All Forests	92.6%	94.0% ± 0.5%	91.2% ± 0.7%

### Per-continent accuracy

Table 9 summarizes overall accuracy and per-class F1-scores broken down by continent, while Table 10 reports the full user’s accuracy, producer’s accuracy, and their standard errors for each class–continent combination, along with the number of predicted and reference samples.

**Table 9:** Per-continent classification accuracy for the six-class Forest Typology scheme.

	*N*	OA ± SE	Primary F1	NatReg F1	Planted F1	Plantation F1	TreeCrop F1	Other F1
Asia	2,847	90.2 ± 0.6	82.7	70.4	46.4	52.1	69.7	97.0
Africa	1,320	90.5 ± 0.8	78.0	62.5	—	80.0	57.1	96.2
N. America	1,345	90.2 ± 0.7	77.3	78.1	39.4	78.0	50.2	96.4
S. America	1,189	90.7 ± 0.9	92.6	75.1	—	82.1	59.7	94.9
Europe	1,167	88.5 ± 1.1	76.1	79.5	69.7	36.3	40.6	96.5
Austr. & Oc.	277	89.4 ± 1.9	65.8	69.2	—	93.1	6.8	95.1
**Global**	**8,190**	**90.2** ± **0.3**	**82.9**	**72.6**	**58.2**	**66.7**	**62.1**	**96.4**

*N* is the number of validation samples. OA = overall accuracy. All values in %.

**Table 10:** Detailed per-continent and per-class accuracy for the six-class Forest Typology scheme.

Continent	Class	*N*_pred_	*N*_ref_	UA ± SE	PA ± SE	F1
Africa	Primary	218	226	80.6 ± 2.6	75.5 ± 3.4	78.0
	NatReg	147	199	73.6 ± 3.7	54.4 ± 3.8	62.5
	Plantation	17	18	82.4 ± 9.2	77.8 ± 9.7	80.0
	TreeCrop	136	148	90.6 ± 2.4	41.7 ± 6.1	57.1
	Other	802	728	93.6 ± 0.8	99.0 ± 0.2	96.2
Asia	Primary	472	371	74.1 ± 2.0	93.6 ± 1.4	82.7
	NatReg	420	583	84.5 ± 2.2	60.3 ± 2.6	70.4
	Planted	224	135	43.9 ± 3.3	49.2 ± 9.2	46.4
	Plantation	236	287	62.7 ± 6.4	44.5 ± 4.4	52.1
	TreeCrop	547	526	75.2 ± 3.7	64.9 ± 6.2	69.7
	Other	948	945	96.7 ± 0.6	97.2 ± 0.3	97.0
Australia & Oceania	Primary	52	44	66.0 ± 6.5	65.5 ± 8.8	65.8
	NatReg	42	55	83.5 ± 6.2	59.1 ± 8.0	69.2
	Plantation	29	29	93.1 ± 4.7	93.1 ± 4.7	93.1
	TreeCrop	2	5	100.0 ± 0.0	3.5 ± 3.3	6.8
	Other	152	144	93.0 ± 2.1	97.4 ± 0.8	95.1
Europe	Primary	39	28	67.1 ± 7.6	87.9 ± 5.7	76.1
	NatReg	288	334	79.8 ± 3.0	79.2 ± 2.6	79.5
	Planted	418	371	74.1 ± 2.2	65.8 ± 4.4	69.7
	Plantation	24	32	48.5 ± 10.1	29.0 ± 7.2	36.3
	TreeCrop	48	47	62.8 ± 6.9	30.0 ± 13.9	40.6
	Other	350	355	96.3 ± 1.0	96.7 ± 0.7	96.5
North America	Primary	196	171	71.4 ± 3.2	84.4 ± 2.4	77.3
	NatReg	282	289	80.1 ± 2.6	76.3 ± 2.9	78.1
	Planted	53	91	84.9 ± 4.9	25.6 ± 4.7	39.4
	Plantation	212	198	82.5 ± 2.6	73.9 ± 7.6	78.0
	TreeCrop	37	44	94.6 ± 3.7	34.1 ± 9.9	50.2
	Other	565	552	96.5 ± 0.6	96.3 ± 0.6	96.4
South America	Primary	339	302	88.2 ± 1.7	97.5 ± 1.2	92.6
	NatReg	193	264	85.6 ± 2.7	66.9 ± 3.2	75.1
	Plantation	130	137	84.6 ± 3.1	79.6 ± 3.4	82.1
	TreeCrop	54	44	58.7 ± 12.1	60.7 ± 14.0	59.7
	Other	473	439	94.2 ± 1.1	95.5 ± 0.8	94.9
**Global**	**Primary**	**1,326**	**1,148**	**77.9** ± **1.1**	**88.5** ± **1.1**	**82.9**
	**NatReg**	**1,375**	**1,736**	**81.1** ± **1.2**	**65.8** ± **1.4**	**72.6**
	**Planted**	**701**	**602**	**65.1** ± **1.8**	**52.6** ± **3.7**	**58.2**
	**Plantation**	**650**	**702**	**74.4** ± **3.1**	**60.4** ± **3.2**	**66.7**
	**TreeCrop**	**831**	**818**	**76.9** ± **2.8**	**52.1** ± **4.2**	**62.1**
	**Other**	**3,307**	**3,184**	**95.4** ± **0.4**	**97.3** ± **0.2**	**96.4**

${N}_{{\rm{pred}}}$/${N}_{{\rm{ref}}}$: samples predicted as / referenced as each class. UA = user’s accuracy, PA = producer’s accuracy.

Overall accuracy is consistent across continents, ranging from 88.5% (Europe) to 90.7% (South America). However, per-class performance varies substantially. Primary forest F1-scores are highest in South America (92.6%), where large contiguous tropical forests are well-represented, and lowest in Australia (65.8%), where the small validation sample (N=277) limits statistical power. Naturally regenerating forest achieves the most uniform performance across continents (62.5–79.5% F1).

Rare class–continent combinations should be interpreted with caution. For example, tree crops in Australia (${N}_{{\rm{ref}}}=5$), planted forest in Africa (${N}_{{\rm{pred}}}=0$), and plantation forest in Europe (${N}_{{\rm{ref}}}=32$) yield unstable estimates with large standard errors.

### Comparison to other forest maps

We evaluate the Forest Typology map against other publicly available global forest maps on the same GVFT validation dataset. For binary forest/non-forest classification (Table 11), Forest Typology yields the highest overall accuracy among the compared products: JRC GFC2020^19^, ESA WorldCover 2020^32^, and ALOS PALSAR FNF4^53^. For the four-class forest type scheme comparable to JRC GFT classes (Table 12), Forest Typology improves overall accuracy by 3.1 percentage points over JRC GFT2020, with consistent gains across all classes—most notably for primary forest (+6.0 pp F1) and planted forest (+8.6 pp F1).

**Table 11:** Forest/non-forest classification accuracy of the Forest Typology map and other global forest maps evaluated on the GVFT dataset.

Map	OA	OA SE	Forest F1	Non-forest F1
JRC GFC2020 (v3)	93.6%	0.3%	89.7%	95.4%
JRC GFC2020 (v2)	92.5%	0.3%	88.5%	94.5%
JRC GFC2020 (v1)	91.8%	0.3%	87.3%	94.0%
ESA WorldCover 2020	91.6%	0.3%	86.8%	93.8%
ALOS PALSAR FNF4	86.9%	0.5%	80.9%	90.1%
**ForTy 2020**	**95.5%**	0.3%	**92.6%**	**96.8%**

**Table 12:** Four-class forest type accuracy (JRC GFT scheme: primary, naturally regenerating, planted, other) evaluated on the GVFT dataset. F1-scores reported per class.

Map	OA	OA SE	Primary F1	NatReg F1	Planted F1	Other F1
JRC GFT2020 (v1)	87.8%	0.4%	76.9%	66.7%	57.2%	95.3%
**ForTy 2020**	**90.9%**	0.3%	**82.9%**	**72.3%**	**65.8%**	**96.7%**

### Spatial agreement with independent datasets

We also compare the Forest Typology map spatially against three independent datasets that characterize well-defined subsets of the forest typological spectrum: Intact Forest Landscapes (IFL) 2020^14,39^, the Primary Humid Tropical Forests product^13^, and the SBTN Natural Lands map^54^. For each comparison, we report the fraction of the external product’s area that falls within expected Forest Typology classes in Table 13. These are consistency checks rather than accuracy assessments: disagreements may originate from either map.

**Table 13:** Spatial agreement between the Forest Typology map and three independent datasets.

External dataset	Expected Forest Typology classes	Agreement
IFL 2020	Primary *OR* NatReg *OR* Other	99.998%
Primary Humid Tropical^†^	Primary	90.4%
Primary Humid Tropical^†^	Primary *OR* NatReg	99.1%
SBTN Natural Lands	Primary *OR* NatReg *OR* Other	98.9%

Each row reports the percentage of the external dataset’s area classified into expected Forest Typology classes.

^†^After excluding tree cover loss 2001–2020^7^.

### Intact Forest Landscapes (IFL)

IFL delineates contiguous blocks of primary forest and associated natural ecosystems larger than 500 km^2^ with no signs of significant human activity^14^. Within the IFL 2020 extent, 99.998% of the area is classified by the Forest Typology map as either primary forest, naturally regenerating forest, or other land (non-forest portions of IFL such as water, rock, and ice). Only 0.002% of the IFL area falls within the planted, plantation, or tree crop classes, confirming strong spatial consistency between the two products for intact natural forest areas.

### Primary Humid Tropical Forests (PHTF)

The pan-tropical primary humid forests were mapped circa 2001^13^. We exclude all tree cover loss from 2001 to 2020^7^ to approximate the 2020 primary forest extent. Of this adjusted area, 90.4% is classified as primary forest by the Forest Typology map, and 99.1% as either primary or naturally regenerating forest. The remaining 0.9% largely corresponds to non-forest land cover.

The 9.6% of primary humid tropical forest area that the Forest Typology map classifies as naturally regenerating rather than primary can be attributed to various factors: (a) PHTF may include mature secondary forests that were not recognizable from the used Landsat imagery; (b) omissions in the tree cover loss data that was used to propagate the data from 2001 to 2020; and (c) a spatial buffering effect in the Forest Typology primary forest class, inherited from the training data. The global forest management (GFM) dataset^18^, which provides the primary training labels for the primary versus naturally regenerating distinction, was collected through visual interpretation of high-resolution imagery. In this process, interpreters tended to classify mature forest stands in proximity to roads, settlements, or agricultural land as naturally regenerating rather than primary, even when the canopy appeared undisturbed. The Forest Typology model learned and generalized this spatial pattern from the training data, resulting in a conservative primary forest class that systematically excludes forest edges near human infrastructure. This is a property of the training data labeling convention rather than an explicit model design choice.

### SBTN Natural Lands

The Science Based Targets Network (SBTN) Natural Lands map^54^ identifies areas of natural land cover globally. Within the SBTN natural land extent, 98.9% is classified by the Forest Typology map as primary forest, naturally regenerating forest, or other land. The 1.1% classified as planted, plantation, or tree crop may reflect either Forest Typology commission errors in managed landscapes or areas where the SBTN product extends its natural land designation into land with some degree of management.

### Area estimates by forest type

We report area estimates using two complementary approaches: sample-based estimates derived from the stratified validation sample, following the methodology of^51,52,55^, and map-based estimates obtained by pixel counting. Table 14 compares both approaches at the continental level for merged forest type classes. The map-based stratum areas per continent, used as weights for the stratified area estimator, are listed in Table 15. Detailed per-class breakdowns by continent are provided in Tables 16 and 17.

**Table 14:** Sample- and map-based area estimates per continent (Mha, CI_95_ = 1.96 × SE).

Continent	*N*	Forest		Natural		Planted		TreeCrop	
		Sample ± CI_95_	Map	Sample ± CI_95_	Map	Sample ± CI_95_	Map	Sample ± CI_95_	Map
Africa	1,320	694.6 ± 53.1	601.2	692.3 ± 53.1	598.4	2.2 ± 0.8	2.8	51.5 ± 13.3	27.4
Asia	2,847	1321.5 ± 59.0	1309.2	1237.5 ± 56.7	1242.7	84.0 ± 16.3	66.5	106.1 ± 20.9	84.3
Aust. & Oc.	277	167.2 ± 30.9	144.2	163.6 ± 30.8	140.9	3.6 ± 0.9	3.3	7.9 ± 12.2	0.3
Europe	1,167	373.2 ± 26.2	374.6	284.6 ± 23.5	293.9	88.6 ± 11.6	80.7	12.2 ± 10.7	5.4
N. America	1,345	739.5 ± 45.0	744.8	679.6 ± 43.3	712.8	59.8 ± 12.4	32.0	10.2 ± 5.6	4.1
S. America	1,189	879.5 ± 36.7	862.6	864.9 ± 36.7	848.9	14.6 ± 1.7	13.7	9.9 ± 4.8	8.6
Global	8,190	4141.7 ± 105.3	4036.7	3890.4 ± 102.6	3837.6	251.3 ± 23.9	199.0	195.9 ± 31.0	130.2

*N* is the number of consensus validation samples.

**Table 15:** Map-based stratum areas by continent (in Mha).

	Africa	Asia	Australia & Oc.	Europe	North America	South America
Primary forest	321.98	725.70	90.63	65.10	337.09	583.18
Naturally regenerating forest	330.83	513.52	58.93	216.48	386.11	292.07
Planted forest	0.22	83.59	0.01	103.12	17.73	0.00
Plantation forest	5.54	42.55	7.21	5.39	31.87	25.49
Tree crops and agroforestry	19.25	82.42	0.42	18.93	6.17	18.62
Other land	1842.11	2740.40	623.04	491.62	1377.39	670.04
Potential forest	491.48	282.84	42.05	83.04	241.22	198.59

**Table 16:** Sample-based area estimates per forest type class by continent (Mha ± CI_95_, CI_95_ = 1.96 × SE).

	*N*	Primary	NatReg	Planted	Plantation	TreeCrop	Other
Africa	1,320	394.1 ± 38.8	298.2 ± 36.2	0.1 ± 0.2	2.1 ± 0.8	51.5 ± 13.3	2244.2 ± 43.1
Asia	2,847	661.1 ± 38.1	576.4 ± 42.0	38.4 ± 14.0	45.5 ± 8.5	106.1 ± 20.9	3063.0 ± 40.8
Austr. & Oc.	277	90.8 ± 25.0	72.8 ± 18.1	0.0 ± 0.0	3.6 ± 0.9	7.9 ± 12.2	636.4 ± 29.2
Europe	1,167	52.7 ± 12.8	231.9 ± 19.7	84.5 ± 11.5	4.1 ± 1.4	12.2 ± 10.7	608.5 ± 16.5
N. America	1,345	305.8 ± 28.6	373.9 ± 32.5	35.8 ± 11.3	24.0 ± 5.0	10.2 ± 5.6	1677.3 ± 27.7
S. America	1,189	550.4 ± 23.6	314.5 ± 28.1	0.3 ± 0.3	14.3 ± 1.6	9.9 ± 4.8	880.9 ± 25.3
**Global**	**8,190**	**2038.4** ± **70.7**	**1852.0** ± **74.3**	**160.1** ± **22.0**	**91.1** ± **9.4**	**195.9** ± **31.0**	**9146.3** ± **77.0**

*N* is the number of consensus validation samples.

**Table 17:** Map-based forest area estimates per forest type class by continent (Mha).

	Primary	NatReg	Planted	Plantation	TreeCrop	Other
Africa	353	246	0	3	27	2362
Asia	859	383	40	26	84	3097
Austr. & Oc.	87	54	0	3	0	667
Europe	62	232	77	3	5	614
N. America	366	347	11	21	4	1678
S. America	604	245	0	14	9	899
**Global**	**2330**	**1508**	**129**	**70**	**130**	**9317**

Globally, the sample-based estimate of total forest area (classes 1–4) is 4,142 Mha ±105 CI_95_, compared to map-based estimate of 4,037 Mha (Table 14). Natural forest (primary + naturally regenerating) accounts for the vast majority at 3,890 Mha (sample-based) versus 3,838 Mha (map-based). Within this, primary forest contributes 2,038 Mha and naturally regenerating forest 1,852 Mha (see Table 16), together covering approximately 29% of the world’s land area. Differences between sample-based and map-based estimates reflect the known tendency of pixel counting to overestimate classes with high commission error and underestimate those with high omission error; sample-based estimates correct for this bias^51^. Planted forests (251 Mha sample-based, 199 Mha map-based) are comparatively small but regionally significant. Tree crops and agroforestry cover an estimated 196 Mha (sample-based), predominantly in Asia.

The continental distribution reveals distinct patterns (Table 14). Asia holds the largest natural forest area (1,238 Mha), followed by South America (865 Mha), Africa (692 Mha), and North America (680 Mha). Planted forests are concentrated in Europe (89 Mha) and Asia (84 Mha). Africa shows the largest discrepancy between sample-based and map-based forest estimates (695 vs. 601 Mha), suggesting substantial forest omission in the map. Standard errors are generally small relative to the area estimates, reflecting adequate sample sizes across most continent–class combinations, though some rare classes (e.g., planted forest in Africa and tree crops in Australia) have wide confidence intervals due to limited validation samples. Per-country area estimates for the countries with the largest forest areas are provided in Table 18.

**Table 18:** Sample- and map-based area estimates (Mha) with 95% Confidence Intervals (1.96 × SE) for countries with largest forest areas.

Country	N	Forest		Natural		Planted		TreeCrop	
		Sample ± CI_95_	Map	Sample ± CI_95_	Map	Sample ± CI_95_	Map	Sample ± CI_95_	Map
Russia	911	858.1 ± 25.4	853.6	839.3 ± 26.5	841.1	18.1 ± 7.2	12.5	0.2 ± 0.3	0.1
Brazil	726	479.3 ± 18.8	473.8	469.8 ± 18.8	465.7	10.6 ± 2.9	8.0	4.4 ± 2.4	3.8
Canada	462	419.1 ± 22.3	396.6	395.7 ± 23.8	389.9	21.2 ± 8.8	6.7	0.0 ± 0.0	0.0
USA	687	294.0 ± 14.6	315.1	250.7 ± 16.3	282.5	41.4 ± 8.7	32.6	1.3 ± 0.5	1.3
China	816	202.9 ± 14.3	193.6	142.9 ± 9.7	151.5	61.0 ± 15.0	42.2	22.6 ± 18.0	5.3
DRC	166	161.9 ± 5.6	157.7	161.9 ± 5.6	157.7	0.0 ± 0.0	0.1	4.6 ± 4.5	0.8
Australia	233	158.6 ± 27.2	132.7	156.8 ± 27.3	130.9	1.7 ± 0.7	1.8	6.4 ± 12.2	0.2
Indonesia	345	125.1 ± 4.8	123.4	122.7 ± 4.8	120.9	2.3 ± 0.6	2.5	29.2 ± 4.8	29.3
Mexico	121	53.9 ± 6.6	59.7	53.9 ± 6.6	59.6	0.0 ± 0.0	0.1	2.7 ± 2.5	1.4
India	142	53.1 ± 10.3	51.2	50.1 ± 11.1	50.6	2.8 ± 3.6	0.6	9.4 ± 6.0	9.4
Sweden	107	26.0 ± 3.2	25.6	9.3 ± 3.6	12.1	16.3 ± 4.2	13.5	0.0 ± 0.0	0.0
Viet Nam	100	15.8 ± 1.2	14.3	13.2 ± 1.1	11.9	2.6 ± 0.6	2.4	4.7 ± 1.7	3.5

Several continent-level discrepancies between sample-based and map-based area estimates reveal systematic patterns in the map’s classification behavior. In Asia, the map assigns 859 Mha to primary forest, whereas the sample-based estimate is 661 Mha (Tables 16, 17). Conversely, naturally regenerating forest is underestimated by the map (383 Mha) relative to the sample-based estimate (576 Mha). The total natural forest area is similar under both approaches (map: 1,243 Mha; sample: 1,238 Mha), indicating that the discrepancy is largely a redistribution between primary and naturally regenerating forest rather than an overall forest area bias. This pattern is most pronounced in Russia (Table 18), where vast boreal forests are difficult to classify along the primary–naturally regenerating gradient from satellite imagery alone. The distinction between undisturbed primary boreal forest and forests with historical or low-intensity management is inherently ambiguous in these regions.

In Africa, the sample-based total forest estimate (695 Mha) exceeds the map-based estimate (601 Mha) by approximately 94 Mha, the largest absolute discrepancy among all continents. This gap likely reflects the omission of sparse woodland and savanna forests near the FAO canopy cover threshold (10%), which are widespread across the Sahel, Miombo, and other dry woodland ecosystems.

In Australia and Oceania, the sample-based tree crop estimate (7.9 ± 12.2 Mha) has a 95% confidence interval wider than the point estimate itself, reflecting the very small number of tree crop validation samples in this region. The map-based estimate is 0.3 Mha. While the discrepancy is large in relative terms, both estimates are small in absolute terms, and the wide confidence interval indicates that the sample-based estimate is not statistically distinguishable from zero at conventional significance levels.

### Per-country forest proportions and the 30 × 30 biodiversity target

**Fig. 8:**
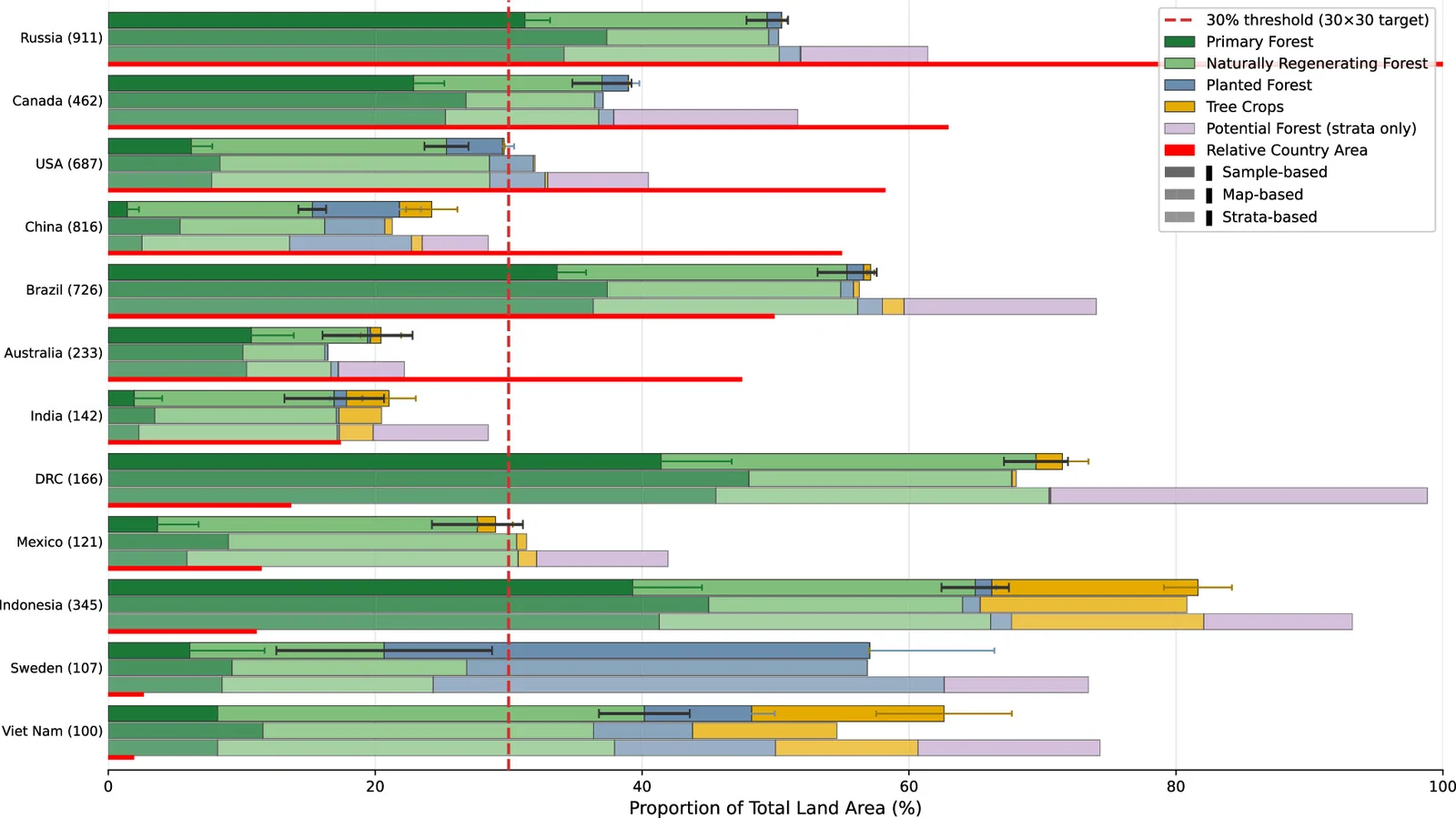
Forest and tree cover by type as estimated proportions of total country land area for countries with at least 100 validation samples. The 4 bars per country show: (1) sample-based estimates with 95% confidence intervals at each class boundary, (2) map-based estimates, (3) strata proportions, (4) relative land area (relative to the largest country). The dashed red line marks the 30% threshold of the 30 × 30 biodiversity target. Numbers in parentheses indicate the number of validation samples per country. The thick black error bar indicates sample-based estimates CI_95_ for all natural forests (primary and naturally regenerating).

Figure 8 presents per-country forest composition as a proportion of total land area for countries with at least 100 validation samples. For each country, four horizontal bars are shown (from top to bottom): (1) *sample-based estimates*, derived from the stratified validation sample following^51^, with 95% confidence intervals at each class boundary; (2) *map-based estimates*, obtained by pixel counting from the Forest Typology classification map; (3) *strata-based proportions*, reflecting the areas assigned to each stratum during the stratified sampling design, including a “potential forest” stratum that captures areas not classified as forest but considered likely to contain forest; and (4) a thin red bar indicating the *relative country land area*, normalized so that the largest country (Russia) reaches 100%, providing visual context for absolute scale.

These per-country proportions complement the absolute area estimates in Table 18 by normalizing for country size, enabling direct comparison of forest composition across nations of vastly different extents. The 95% confidence intervals reflect the statistical uncertainty of the sample-based estimates; however, for countries with few validation samples, confidence intervals may be artificially narrow because the stratified estimator cannot capture classification variability from a small, homogeneous sample—estimates for such countries should be interpreted with particular caution.

The per-country analysis reveals substantial variation in forest composition. Among the twelve countries with at least 100 validation samples (Fig. 8), countries such as the DRC, Indonesia, and Brazil retain the highest natural forest proportions relative to total land area. Sweden illustrates a case where total forest cover is high but predominantly planted: approximately two-thirds of Sweden’s forest area is classified as planted or plantation, with natural forest covering only about a fifth of the country’s land area. For such countries, the distinction between natural and planted forest is especially policy-relevant: total tree cover may appear adequate, yet the natural forest component that supports the bulk of biodiversity may be critically low. The ability to separately quantify primary, naturally regenerating, and planted forest at the national level provides the thematic resolution needed for evidence-based conservation prioritization under the Kunming-Montreal 30 × 30 framework.

The ecological significance of the naturally regenerating forest class varies across biomes and regions. In tropical systems, secondary forests can recover substantial species richness and biomass within decades, playing a critical role in landscape-level biodiversity conservation and carbon sequestration^29,56^. In temperate and boreal regions, post-disturbance regeneration may follow slower successional trajectories, with community composition that differs markedly from the pre-disturbance state. The per-country breakdown in Fig. 8 reflects these regional differences: high proportions of naturally regenerating forest in tropical countries often indicate post-agricultural recovery, while in temperate regions they may reflect post-logging or post-fire regrowth.

## Limitations

### Classification boundaries

The FAO-based definitions underlying the Forest Typology classification rely on management intent and history, which are not directly observable from single-year satellite imagery. The model infers these categories from proxy indicators—spectral signatures, canopy structure, and spatial context—introducing irreducible uncertainty at class boundaries. This is most pronounced for the distinction between primary forest and naturally regenerating forest, where mature secondary forests can be spectrally and structurally indistinguishable from undisturbed primary forests. In addition, the primary forest class boundary is conservative near human infrastructure, a spatial buffering effect inherited from the labeling conventions of the GFM training data^18^, as discussed above. Planted forests exhibit the lowest classification accuracy among all forest classes, as they overlap spectrally with naturally regenerating forests at one end and plantation forests at the other. Mixed agroforestry systems—where tree crops such as cocoa or coffee grow beneath a native canopy—remain among the most challenging classes; while this study represents a significant advance, shaded agroforests can still be misclassified as natural forest in some regions.

### Definitional ambiguities

The FAO forest definition includes land with trees “able to reach [the] thresholds in situ” (>0.5 ha, >5 m height, >10% canopy cover), encompassing temporarily unstocked areas such as recently harvested plantations awaiting replanting or forests in early post-disturbance recovery. Because the map is derived from a single year of satellite observations, such areas may appear as non-forest and be classified as Other Land, even though they remain forest under the FAO definition by management intent. Similarly, the 40-year rotation threshold used to distinguish planted forest from plantation forest is an operational proxy; the FAO and EUDR definitions rely on management intensity criteria that are not directly observable from imagery. The Other Land class subsumes other wooded land (OWL), which is a distinct EUDR category. Also, the Tree Crops and Agroforestry class (Class 5) groups together systems spanning a wide gradient of structure and ecological function—from intensive monoculture plantations (e.g., oil palm estates, fruit orchards) to highly heterogeneous, biodiversity-rich agroforestry systems (e.g., shaded cocoa, complex agroforests). While this aggregation is necessary for operational global mapping and regulatory alignment, one should note that the ecological and biodiversity implications of this class vary substantially across landscapes, particularly in tropical regions.

### Degraded and selectively logged forests

Forests degraded by selective logging, wildfire, fragmentation, or informal management present a particular classification challenge. Such forests may retain sufficient canopy cover to appear as forest in satellite imagery, yet they might no longer be ecologically primary. Because the FAO definition of primary forest requires “no clearly visible indications of human activities”, degraded forests can fall on either side of the Primary–NatReg boundary depending on whether the disturbance is detectable from available imagery and reference data.

### Temporal constraints

The map represents land cover as of the year 2020, derived from multi-temporal satellite composites that aggregate observations across an entire calendar year. Changes occurring late in the compositing period may not be fully captured, introducing a temporal lag of up to several months. Following wildfire events, distinguishing the pre-fire forest type from subsequent regeneration or land-use conversion is particularly challenging from a single-year observation window. Consequently, recently burned forests may carry higher classification uncertainty, and burned areas may be temporarily misclassified as “other land” or as a different forest type during the early stages of recovery. In practice, forest areas affected by fire in 2020 should be interpreted with caution, as their classification may not reflect the pre-disturbance forest type.

### Spatial resolution

At 10 m resolution, mixed pixels at forest-type boundaries may not resolve fine-scale transitions accurately. Sub-pixel heterogeneity, particularly in smallholder landscapes, can reduce classification precision. Isolated tree patches near the FAO forest area threshold (0.5 ha) and areas at the canopy cover boundary (10%) are inherently ambiguous and may be inconsistently classified. Additionally, the model input data’s effective resolution might be worse than 10 m.

### Probability calibration

The per-class relative confidence scores represent ensemble-averaged softmax outputs and have not been post-hoc calibrated. While ensemble averaging generally improves calibration compared to single-model predictions, the confidence scores may not precisely reflect true posterior class membership^45^. Users requiring well-calibrated probabilities for quantitative risk assessment should consider regional recalibration using independent reference data.

## Usage Notes

The Forest Typology v1 map is distributed as a five-band relative confidence scores raster (uint8, values 0–250) covering the five forest and tree cover classes. The “other” class confidence score is computed as $250-{\sum }_{i\mathrm{=1}}^{5}{p}_{i}$. A categorical map is derived by taking the argmax across all six class scores. For binary forest/non-forest masks, the confidence scores for classes 1–4 can be summed, versus the remainder. Similarly, natural or managed forest distinction is obtained by summing classes 1–2 (natural) and 3–4 (managed), respectively.

The confidence scores representation supports custom thresholding for applications with different precision-recall trade-offs:

- To prioritize user’s accuracy (minimizing commission errors, i.e., high confidence that mapped forests are truly of that type), select a higher confidence threshold.
- To prioritize producer’s accuracy (minimizing omission errors, i.e., capturing most of the actual forest of a given type), select a lower threshold.
- To seek a balance, choose a threshold near the intersection of the UA and PA curves (see Fig. 7), or where both accuracies are acceptably high for the application.

We note that the distributed confidence scores are *ensemble-averaged softmax outputs* and may not be perfectly calibrated as true posterior class probabilities. Users are encouraged to evaluate the score distribution in their region of interest and, if necessary, apply domain-specific threshold selection using local validation data^23,46^.

## Data Availability

The Forest Typology v1 map for 2020 at 10 m resolution is available at https://doi.org/10.25452/figshare.plus.32288616, theextracted classification layer is available for download at https://doi.org/10.25452/figshare.plus.32301177, and the GlobalValidation of Forest Types (GVFT) dataset (v0.6) is available at https://doi.org/10.25452/figshare.plus.33093404.

## Code availability

We generated the training dataset and the final map using the *GeeFlow* library (https://github.com/google-deepmind/geeflow), which uses Google Earth Engine^57^ as its computational backend. The code for model training, inference, and evaluation is available in the *JEO* code repository (https://github.com/google-deepmind/jeo).
